# TaNAC29, a NAC transcription factor from wheat, enhances salt and drought tolerance in transgenic *Arabidopsis*

**DOI:** 10.1186/s12870-015-0644-9

**Published:** 2015-11-04

**Authors:** Quanjun Huang, Yan Wang, Bin Li, Junli Chang, Mingjie Chen, Kexiu Li, Guangxiao Yang, Guangyuan He

**Affiliations:** The Genetic Engineering International Cooperation Base of Ministry of Science and Technology, Key Laboratory of Molecular Biophysics of Ministry of Education, College of Life Science and Technology, Huazhong University of Science & Technology (HUST), Wuhan, 430074 China

**Keywords:** Wheat, *Arabidopsis*, NAC, *TaNAC29*, Abiotic stress, ABA-hypersensitive

## Abstract

**Background:**

NAC (NAM, ATAF, and CUC) transcription factors play important roles in plant biological processes, including phytohormone homeostasis, plant development, and in responses to various environmental stresses.

**Methods:**

*TaNAC29* was introduced into *Arabidopsis* using the *Agrobacterium tumefaciens*-mediated floral dipping method. *TaNAC29*-overexpression plants were subjected to salt and drought stresses for examining gene functions. To investigate tolerant mechanisms involved in the salt and drought responses, expression of related marker genes analyses were conducted, and related physiological indices were also measured. Expressions of genes were analyzed by quantitative real-time polymerase chain reaction (qRT-PCR).

**Results:**

A novel NAC transcription factor gene, designated *TaNAC29*, was isolated from bread wheat (*Triticum aestivum*). Sequence alignment suggested that *TaNAC29* might be located on chromosome 2BS. TaNAC29 was localized to the nucleus in wheat protoplasts, and proved to have transcriptional activation activities in yeast. *TaNAC29* was expressed at a higher level in the leaves, and expression levels were much higher in senescent leaves, indicating that *TaNAC29* might be involved in the senescence process. *TaNAC29* transcripts were increased following treatments with salt, PEG6000, H_2_O_2_, and abscisic acid (ABA). To examine *TaNAC29* function, transgenic *Arabidopsis* plants overexpressing *TaNAC29* were generated. Germination and root length assays of transgenic plants demonstrated that *TaNAC29* overexpression plants had enhanced tolerances to high salinity and dehydration, and exhibited an ABA-hypersensitive response. When grown in the greenhouse, *TaNAC29*-overexpression plants showed the same tolerance response to salt and drought stresses at both the vegetative and reproductive period, and had delayed bolting and flowering in the reproductive period. Moreover, *TaNAC29* overexpression plants accumulated lesser malondialdehyde (MDA), H_2_O_2_, while had higher superoxide dismutase (SOD) and catalase (CAT) activities under high salinity and/or dehydration stress.

**Conclusions:**

Our results demonstrate that *TaNAC29* plays important roles in the senescence process and response to salt and drought stresses. ABA signal pathway and antioxidant enzyme systems are involved in TaNAC29-mediated stress tolerance mechanisms.

**Electronic supplementary material:**

The online version of this article (doi:10.1186/s12870-015-0644-9) contains supplementary material, which is available to authorized users.

## Background

Plants are frequently challenged by unfavorable environmental conditions, including extreme temperatures, drought, and high salinity. Upon exposure to harmful environmental conditions, many related genes are induced [1]. Transcription factors (TFs) are one such related gene family. Numerous studies demonstrated that TFs play vital roles in plant gene regulation, either activating or preventing target gene expression [2, 3]. Among TFs families, NAC TFs and their corresponding *cis*-acting sequences act as molecular switches to regulate temporal and spatial gene expression [2, 3].

The NAC superfamily is one of the largest TF families in plants. Most NAC proteins share a highly conserved NAC domain at the N-terminal, and a diversified activation domain at the C-terminal [2]. NAC TFs play a vital role in various plant developmental processes, including leaf senescence, phytohormone homeostasis, and responses to unfavorable environmental stresses [2, 3].

In *Arabidopsis*, overexpression of *ANAC019*, *ANAC055*, *RD26*/*ANAC072*, and *ATAF1*/*ANAC002* confer drought tolerance [4, 5]. Plants overexpressing *ATAF2*/*ANAC081* have a greater susceptibility to *Fusarium oxysporum* [6]. Overexpression of *JUB1*/*ANAC042* and *VNI2*/*ANAC083* delays senescence and enhances resistance to abiotic stresses [7, 8]. In rice (*Oryza sativa*), overexpression of *OsNAC5*, *OsNAC9*, and *OsNAC10* significantly enlarges roots, and thereby enhances tolerance to drought stress, furthermore, these transgenic rice plants produce a higher grain yield under field conditions [9–11]. *OsNAC6* overexpression in rice enhances tolerances to salt, drought, and low temperature stresses, but in this case the transgenic rice exhibits low grain yield and growth retardation [12]. Overexpressing *OsNAC045* in rice enhances salt and drought tolerance [13]. When the rice stress-responsive *NAC* gene *SNAC1* was introduced into rice and wheat, the transgenic plants displayed significantly enhanced tolerances to multiple abiotic stresses [14, 15]. Kaneda et al. [16] revealed that overexpression of *OsNAC4* leads to hypersensitive cell death, whereas, in *OsNAC4* knock-down transgenic lines, hypersensitive cell death is significantly reduced. *OsNAC122* and *OsNAC131* proteins are involved in the response to infection by *Magnaporthe grisea*, and may play a role in the phytohormone-mediated signaling pathway [17].

Compared with *Arabidopsis*, rice, and other species, there have been fewer investigations into NAC in wheat. In bread wheat (*Triticum aestivum*), transgenic lines overexpressing *TaNAC69* produce more biomass in the shoot and root when grown under stress-inducing conditions [18]. Overexpression of *TaNAC2*, *TaNAC2a*, and *TaNAC67* in plants improves tolerances to low temperature, high salinity, and drought stresses [19–21]. Quantitative real-time polymerase chain reaction (qRT-PCR) assays suggested that *TaNAC8*, *TaNAC4*, *TtNAMB-2*, and *TaNAC69-1* participate in responses to various biotic and abiotic stresses [22–24]. Overall, these studies demonstrated that the factors mostly affecting expression of NAC genes are salt, drought, and extreme temperatures; and several NAC genes are simultaneously co-expressed in a developmental/organ-specific way.

In this study, a novel NAC transcription factor gene *TaNAC29* was cloned from wheat. Gene expression pattern analysis demonstrated that *TaNAC29* was upregulated by high salinity, dehydration, ABA, and H_2_O_2_ treatments. *TaNAC29* enhanced tolerance to high salinity and drought stress in transgenic *Arabidopsis*, and exhibited an ABA-hypersensitive response. Morphological assays revealed that overexpression of *TaNAC29* delayed bolting and flowering. Our results provide evidence that *TaNAC29* participates in the ABA signal pathway, and plays important roles in stress responses and developmental processes.

## Results

### *TaNAC29* encodes a plant-specific NAC transcription factor

A novel *NAC* gene was cloned from bread wheat. This gene was designated as *TaNAC29* as it had high homology to *NAC29* from *Aegilops tauschii*. It is established that spontaneous hybridization of the wild grass *Aegilops tauschii* (2*n* = 14; DD) with cultivated wheat *Triticum turgidum* (2*n* = 4*x* = 28; AABB) resulted in *T. aestivum* (2*n* = 6*x* = 42; AABBDD) [25]. Moreover, the *Ae. tauschii* genome has been sequenced, and 1489 TFs in 56 families, including the NAC family, have been identified [25]. The full length cDNA of *TaNAC29* is 1198 bp long with a 1074 bp open reading frame (ORF), and encodes a protein with a predicted relative molecular mass of 38.397 kDa.

Sequence alignment and phylogenetic analysis (Additional file 1: Figures S1A and S2) revealed that *TaNAC29* had 96 % identity to W5BNH0 (EMBL: C116E5668.1) from *T. aestivum*, 92 % identity to HvNAC023 (GenBank: CBZ41159.1) from *Hordeum vulgare*, and 89 % identity to NAC29 (GenBank: EMT28859.1) from *Ae. tauschii*. Additionally, *TaNAC29* had relative high homology with OsNAC10 (GenBank: EAZ40329.1) and ANAC047 (*speedy hyponastic growth*; GenBank: AEE74033.1), demonstrating their biological functions [11, 26]. As there was high identity between *TaNAC29* and W5BNH0, nucleic acid sequence alignment was conducted. This revealed that, including the ORF and untranslated region (UTR), *TaNAC29* was 96.5 % identical to W5BNH0 (Additional file 1: Figure S3). Comparison results indicated that *TaNAC29* and W5BNH0 may be the same gene, and similar to W5BNH0, the novel *TaNAC29* might be located on the 2BS chromosome. To further verify if *TaNAC29* and W5BNH0 were the same gene, a wheat whole-genome survey was performed using the *TaNAC29* sequence, and the DNA sequence with the highest identity to *TaNAC29* detected. Use of a DNA splicing program revealed that, excluding the intron, this cDNA sequence was the same with the W5BNH0 sequence. Therefore, the whole-genome survey indicated that *TaNAC29* gene might be the same gene with W5BNH0. To further examine whether slight differences existed, two *TaNAC* genes were selected from NCBI for a BlastN against EMBL; this revealed that *TaNAC2D* (GenBank: GQ231954.1) and *TaNAC69* (GenBank: DQ022842.1) shared 96.8 and 98 % identity to wheat whole-genome cDNAs, respectively. The slight difference between *TaNAC29* and W5BNH0 may be a result of the different wheat cultivars used.

TaNAC29 contains a typical NAC structure with a conserved NAC domain (amino acids 15–177) including five subdomains (A: 15–35, B: 41–61, C: 70–105, D: 114–142, E: 163–177) consistent with NAC conserved domain characteristics. Four L motifs were identified at the divergent C-terminal region (amino acids 178–357) using the multiple EM for motif elicitation (MEME) tool (Additional file 1: Figure S1A and B). Transactivation activity assays confirmed that TaNAC29 was a transcriptional activator, and the C-terminal region possessed transcriptional activation activity (Additional file 1: Figure S4). Kjaersgaard et al. [27] demonstrated that the L motif is sufficient for transactivation activity of HvNAC013. Therefore, to further investigate the function of the L motif, the transactivation activity of seven truncated versions of TaNAC29 was examined. Among these, six truncated versions containing the L motif had transactivation activity, whereas the TaNAC29_1–233_ fragment without the L motif had no transactivation activity (Additional file 1: Figure S4), suggesting that the L motif plays an important role in transactivation activity. Expression of a TaNAC29-GFP fusion protein in wheat mesophyll protoplasts demonstrated that the green fluorescent protein (GFP) and 4′,6-diamidino-2-phenylindole (DAPI; a nuclear stain marker) were confined to the nucleus (Additional file 1: Figure S5); this is consistent with its function as a transcription regulator. Moreover, PONDR VL3 analysis [27, 28] indicated that the C-terminal region of TaNAC29 was intrinsically disordered (ID) to a large degree (Additional file 1: Figure S1C), suggesting that the protein was largely unfolded in the C-terminal region.

### Expression of *TaNAC29* is upregulated by abiotic stresses and signal molecules

Temporal and spatial expression analyses revealed that *TaNAC29* had relatively higher expression levels in the leaf, stem, flag leaf, and stamen, with the highest expression levels occurring in the leaf. However, *TaNAC29* expressed at very low levels in the root, pistil, embryo, endosperm, coleoptile, and caryopsis (Additional file 1: Figure S6). *TaNAC29* transcripts were much higher in mature senescing leaves than in young green leaves. This suggests that *TaNAC29* might be involved in the senescence process in wheat.

**Fig. 1:**
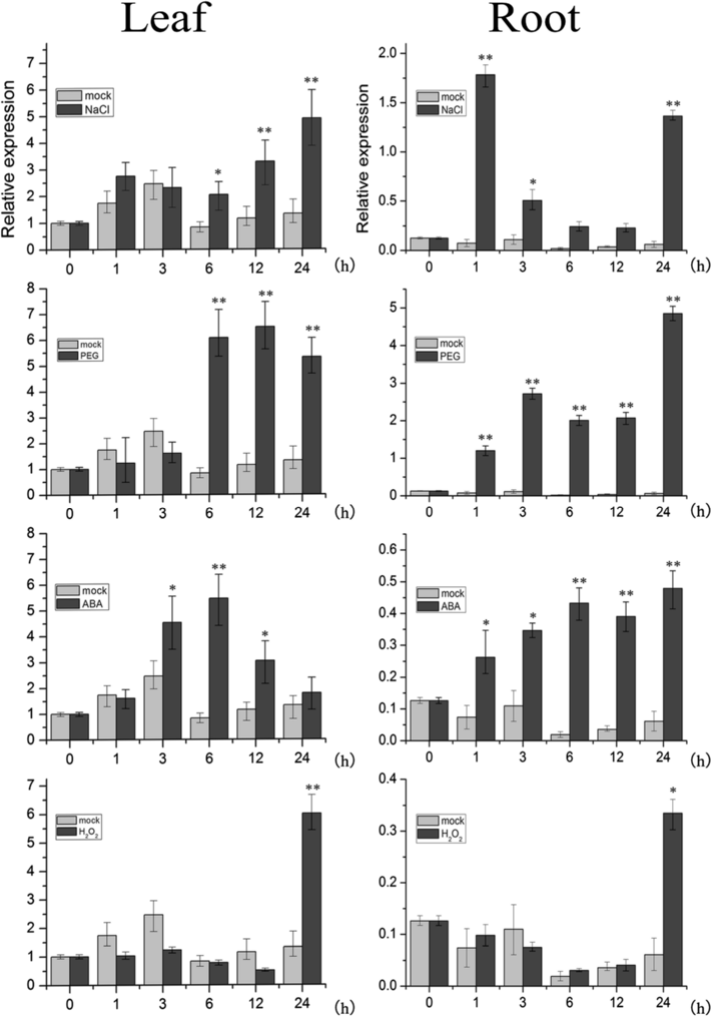
Expression patterns of *TaNAC29* in wheat after stress treatments. Expression patterns of *TaNAC29* in wheat leaves and roots after NaCl, PEG6000, ABA and H_2_O_2_ treatments by qRT-PCR analysis. Leaf and root were collected after different stress treatment. The 2^−ΔΔCT^ method was used in qRT-PCR analysis. Transcript levels were normalized to *TaActin*. Values are means ± SE of three replicates. Asterisks indicate statistically significant differences from mock (*P < 0.05; **P < 0.01). Three independent experiments were performed

To investigate the response of *TaNAC29* to abiotic stresses, *TaNAC29* expression levels were examined following NaCl, PEG6000, H_2_O_2_ and ABA treatments. qRT-PCR analysis revealed that *TaNAC29* was greatly upregulated by NaCl, PEG6000, ABA, and H_2_O_2_ treatments in the leaf, and by NaCl, PEG6000, and ABA treatments in the root; it was only slightly upregulated by H_2_O_2_ in the root (Fig. 1). Interestingly, the expression level increase in the root was stronger than in the leaf following NaCl and PEG treatments, suggesting a close correlation with a relative low organ-specific expression in the root. These qRT-PCR results strongly suggest that *TaNAC29*, like other stress-associated *NAC* genes [4, 11, 18], participates in plant stress responses.

### Salt and drought tolerances of *TaNAC29*-overexpression plants

Transgenic *Arabidopsis* plants were generated to explore the functions of *TaNAC29*. Seven transgenic lines (T_3_) were confirmed through kanamycin resistance analysis. Among these, three overexpression (OE) lines, designated OE1, OE2, and OE3, showed higher *TaNAC29* expression levels by semi-quantitative analysis (Additional file 1: Figure S7). When grown in soil, phenotypes of the *TaNAC29*-overexpression plants were not significantly different from the wild type (WT) at the vegetative phase, but showed delayed bolting and flowering at the reproductive stage under normal growth conditions (Additional file 1: Figure S8). This delayed phenotype was similar to those observed in *JUB1*- and *ATAF1*-overexpressing plants [5, 7].

**Fig. 2:**
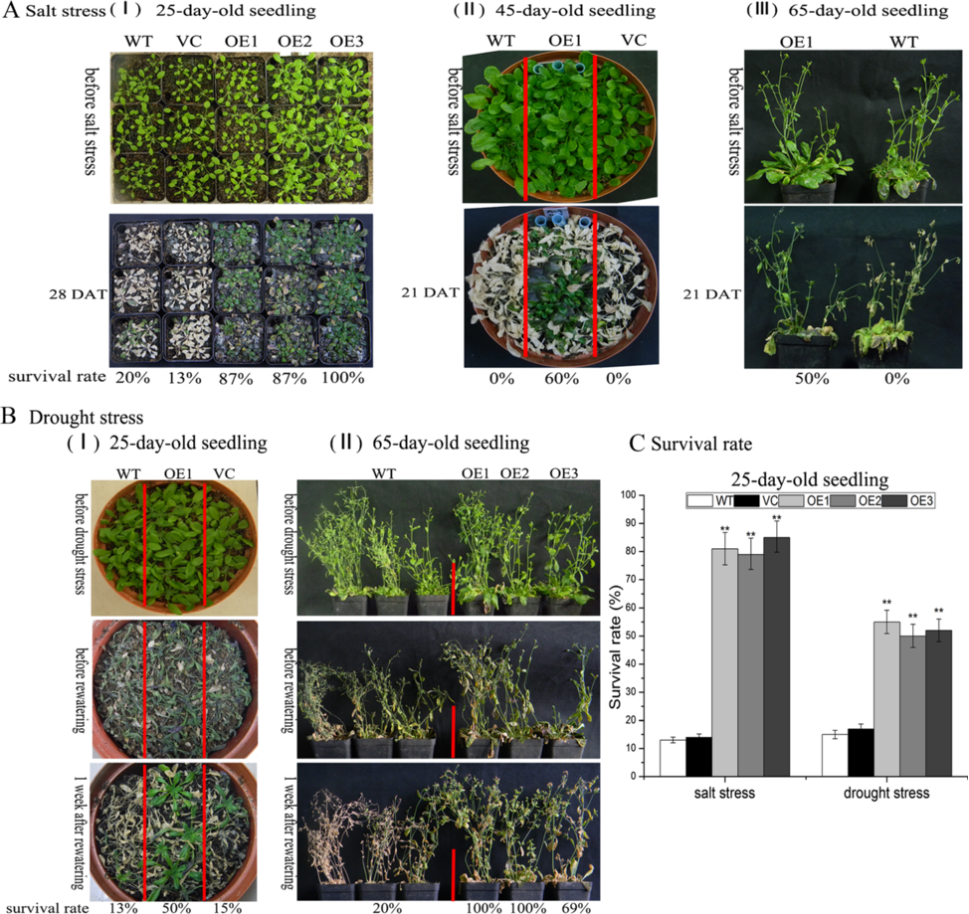
The *TaNAC29*-overexpression (OE) lines have enhanced tolerance to salt and drought stress. **a** Phenotypes of WT, VC (vector control) and OE plants grown on salt soil supplemented with 250 mM NaCl in different growth stage, including (I) 25-day-old seedling, (II) 45-day-old seedling and (III) 65-day-old seedling. **b** Phenotypes of WT, VC and OE plants treated with drought stress at different growth stage, including (I) 25-day-old seedling and (II) 65-day-old seedling. **c** Quantitative analysis of survival rate of 25-day-old seedling after salt and drought stresses. Values are means ± SE of three replicates. Asterisks indicate statistically significant differences from WT (**P < 0.01)

Next, *TaNAC29*-overexpression plants were examined for tolerance to salt stress. Twenty-five-day-old seedlings, grown in soil, were irrigated with 250 mM NaCl solution for 4 weeks (4 w), WT and vector control (VC) plants had a survival rate of ~20 %, while *TaNAC29* overexpressing lines had survival rates of over 80 % (Fig. 2a). When 45-day-old and 65-day-old seedlings were treated for 21 days (21 d) in the same way, all WT and VC plants died, whereas OE1 plants still had a survival rate of over 50 % under these conditions (Fig. 2a). This indicated that overexpression of *TaNAC29* could greatly enhance tolerance to salt stress.

To investigate the drought stress response of the *TaNAC29*-overexpression lines, twenty-five-day-old seedlings at the vegetative phase, were subjected to drought stress through withholding water for 21 d, followed by re-watering for 7 d. Approximately 50 % of OE1 plants recovered from a dying status, whereas the survival rate for both WT and VC plants was ~15 % (Fig. 2b). When 65-day-old seedlings at the reproductive stage were treated in the same way, most transgenic lines still survived, but only 20 % of WT plants were recovered (Fig. 2b). These observations indicated that overexpression of *TaNAC29* could confer resistance to drought stress. Finally, statistical analysis of survival rate of 25-day-old seedling after salt and drought stresses revealed that over 50 % of transgenic plants were still alive, whereas ~85 % of WT and VC plants died (Fig. 2c).

To further verify whether *TaNAC29*-overexpressing plants with enhanced drought stress were associated with transpiration, the phenotype of detached leaves was examined by air-drying in a 25 °C environment. After 5 or 7 h, the leaves of WT and VC had severely curved, whereas the transgenic plant leaves displayed a slightly curled phenotype (Additional file 1: Figure S9A). Water loss rate assays revealed that *TaNAC29-*overexpression lines had a lower rate of water loss at each time point (Additional file 1: Figure S9B), thus rendering *TaNAC29* transgenic plants more tolerant to drought stress.

**Fig. 3:**
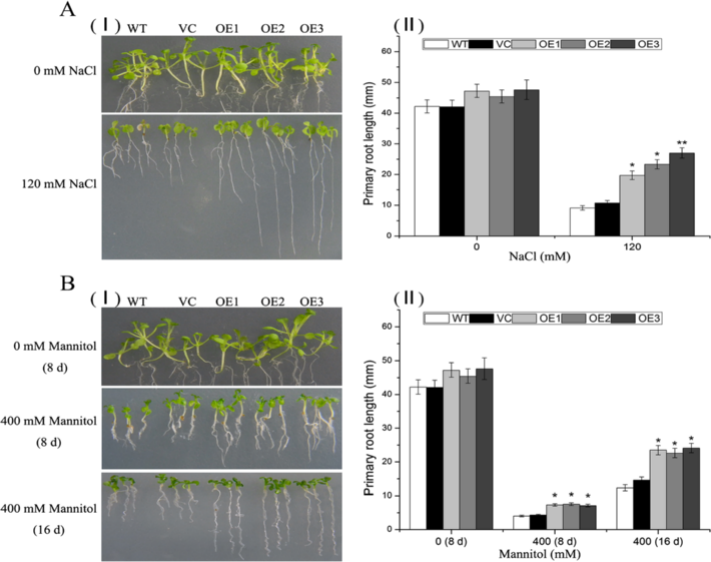
Root length assays of WT, VC (vector control) and overexpression (OE) lines. **a** Phenotypes of WT, VC and OE plants grown for 8 d on medium supplemented with 0 or 120 mM NaCl (I) and primary root length (II). **b** Phenotypes of WT, VC and OE plants grown for 8 and 16 d on medium supplemented with 0 or 400 mM Mannitol (I) and primary root length (II). Values are means ± SE (*n* = 20 to 25 plants) in root length assays. Asterisks indicate statistically significant differences from WT (*P < 0.05, **P < 0.01)

Stress tolerance of plants overexpressing *TaNAC29* was further examined by root length analysis. Transgenic plants had a longer root than WT under drought stress conditions (Additional file 1: Figure S10). When grown on 1/2 Murashige-Skoog (MS) medium containing 120 mM NaCl, the growth of the primary roots of *TaNAC29*-overexpression line seedlings was significantly stronger than observed in WT and VC after 8 d of treatment (Fig. 3a). A dehydration assay indicated that *TaNAC29* transgenic lines exhibited enhanced tolerance on 1/2 MS medium containing 400 mM Mannitol, at both 8 and 16 d after treatment (Fig. 3b). These results further demonstrated that *TaNAC29*-overexpression lines had increased tolerance to salt and drought stresses.

### *TaNAC29*-overexpressing plants exhibit ABA-hypersensitive response

**Fig. 4:**
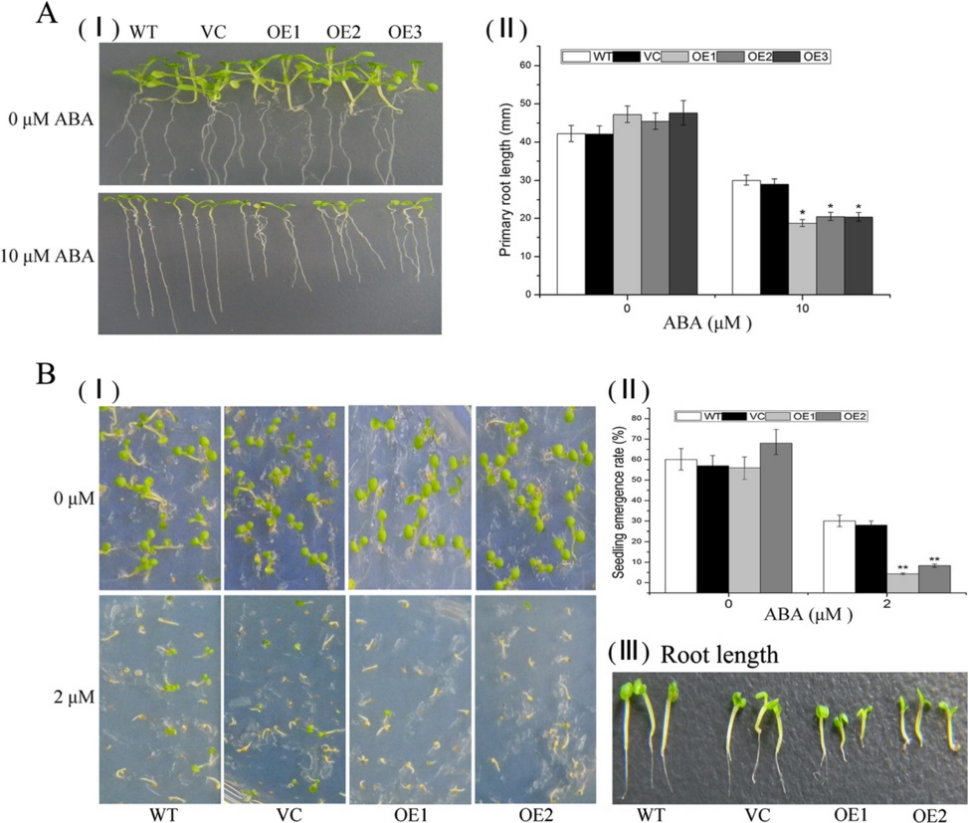
Hypersensitivity of *TaNAC29-*overexpression (OE) lines to ABA. **a** Phenotypes of WT, VC (vector control) and OE plants grown for 8 d on medium supplemented with 0 or 10 μM ABA (I) and primary root length (II). Values are means ± SE (*n* = 20 to 25 plants). Asterisks indicate statistically significant differences from WT (*P < 0.05). **b** Seedlings of WT, VC and *TaNAC29-*overexpression lines observed 8 d after germination on 1/2 MS medium supplemented with 0 or 2 μM ABA (I) and quantitative analysis of seedling emergence rate (II). Values are means ± SE (*n* = 60 to 90 seeds). Asterisks indicate statistically significant differences from WT (**P < 0.01). Comparison of root length of WT, VC and *TaNAC29-*overexpression lines (III). Three independent experiments were performed, each evaluating 60 to 90 seeds

Whether salt and drought tolerances of *TaNAC29*-overexpression line plants was associated with ABA was tested by measuring root length. When grown on 1/2 MS medium containing 10 μM ABA, exogenous ABA inhibited root growth of *TaNAC29*-overexpression line seedlings more severely than observed in WT and VC, suggesting that *TaNAC29* transgenic lines were hypersensitive to ABA (Fig. 4a). Seed germination and seedling emergence (seedling with cotyledon) rate assays were performed to further verify the ABA hypersensitivity of *TaNAC29* transgenic lines. As shown in Fig. 4b−I, when grown on 1/2 MS medium without ABA, there was no significant difference in seedling emergence between WT, VC, and transgenic seeds. However, the seedling emergence rate of WT and VC was higher than those of transgenic seeds on 1/2 MS medium containing 2 μM ABA (Fig. 4b I and II). Additionally, transgenic lines grown on ABA-containing medium had shorter roots than those of WT and VC (Fig. 4b−III). These observations indicated that *TaNAC29* transgenic lines displayed ABA hypersensitivity during post-germination growth, suggesting that *TaNAC29* was positively regulated by ABA.

### Expression of related marker genes under salt and drought stresses

**Fig. 5:**
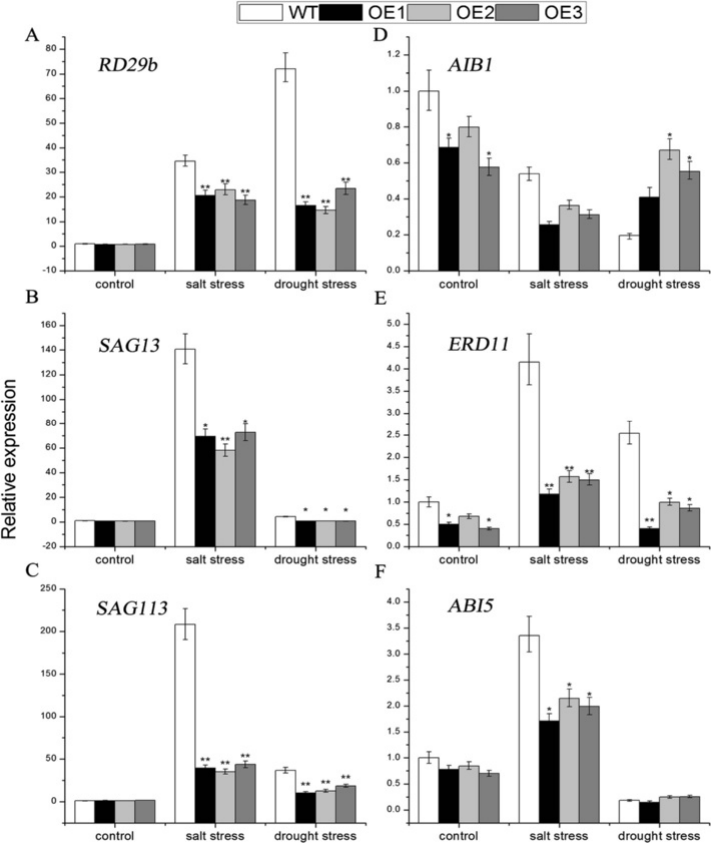
Expression pattern of relevant genes (**a** *RD29b*, **b** *SAG13*, **c** *SAG113*, **d** *AIB1*, **e** *ERD11*, and **f** *ABI5*) in WT and *TaNAC29*-overexpression (OE) plants. Seedlings of WT and OE were treated with 250 mM salt stress for 10 d and drought stress for 17 d, respectively. Total RNAs were extracted from leaves, and qRT-PCR analysis was performed. The 2^−ΔΔCT^ method was used in qRT-PCR analysis. Values are means ± SE of three replicates. Asterisks indicate statistically significant differences from WT (*P < 0.05; **P < 0.01). Three independent biological experiments were performed

To further understand the molecular basis of *TaNAC29* function, the expression levels of related marker genes were analyzed. The expression levels of most related marker genes were significantly lower in *TaNAC29-*overexpression line plants than in WT plants (Fig. 5). The transcript level of *RD29b* (responsive-to-desiccation 29b; an ABA-responsive marker gene) [29] increased 72-fold in WT under drought stress conditions, this was much greater than observed in *TaNAC29-*overexpression line plants (Fig. 5a), indicating that *TaNAC29* might participate in the ABA signal pathway. As indicators for leaf senescence, relative expressions of *SAG13* (senescence-associated gene 13) [30] and *SAG113* (senescence-associated gene 113) [31] were lower in *TaNAC29-*overexpression line plants than in WT plants following salt and drought stresses (Fig. 5b and c), suggesting that *TaNAC29* overexpression delayed leaf senescence effects of abiotic stresses. *AIB1* (ABA-inducible BHLH-type transcription factor/JA-associated MYC2-like1), a negative regulator of jasmonic acid (JA) signaling [32], was only slightly affected by salt and drought stresses (Fig. 5d). The expression levels of *ERD11* (early-responsive-to-dehydration 11; an early responsive-to-dehydration gene) [28] and *ABI5* (ABA-insensitive 5; an ABA signaling regulator) [33] significantly increased in WT plants, but were only slightly changed in plants overexpressing *TaNAC29* (Fig. 5e and f). Taken together, these results demonstrated that *TaNAC29* was involved in regulating the expression of some key ABA signaling regulators and senescence-associated genes.

### Variations of chlorophyll, H_2_O_2_, and malondialdehyde (MDA) content, of electrolytic leakage, and catalase (CAT), superoxide dismutase (SOD), and peroxidase (POD) activities under salt and drought stresses

**Fig. 6:**
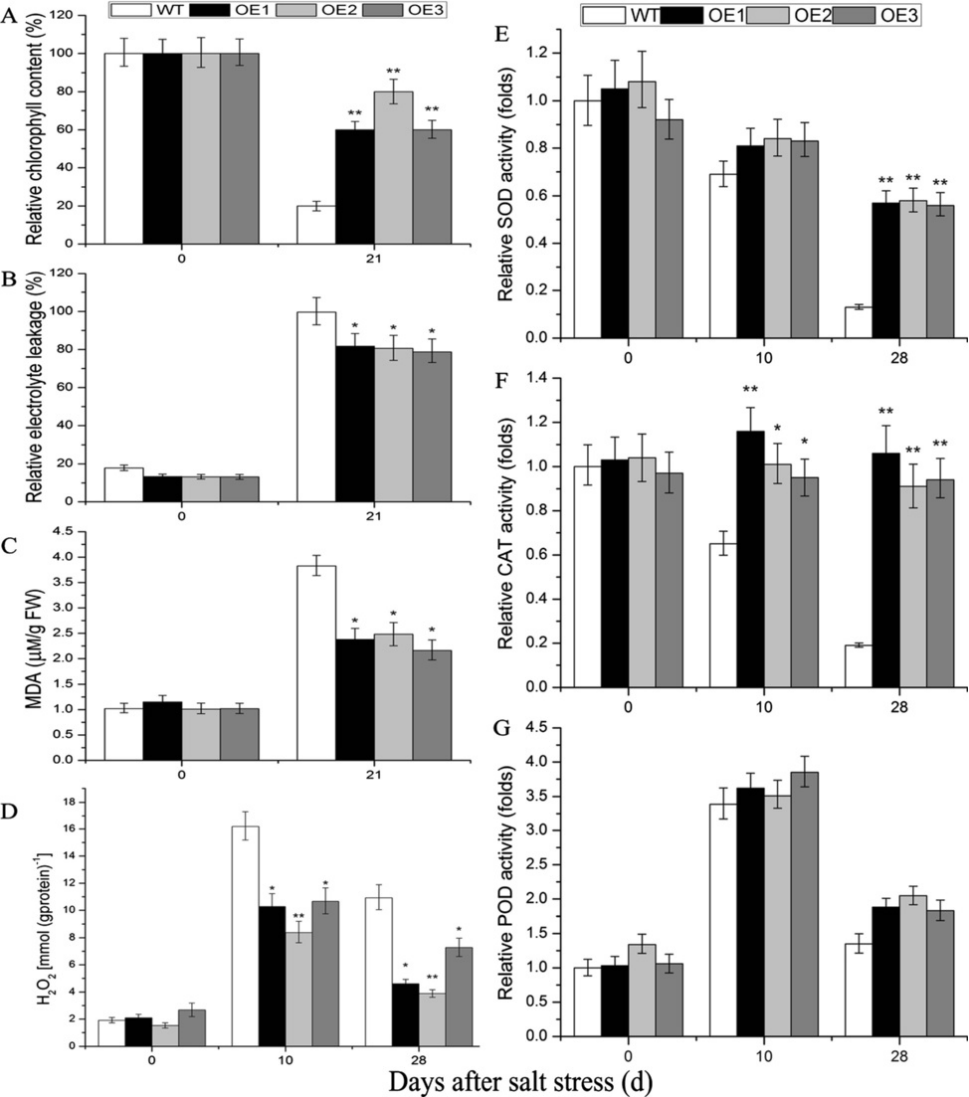
Analysis of physiological indices under salt stress conditions. Analysis of chlorophyll content (**a**), electrolyte leakage (**b**), MDA content (**c**), H_2_O_2_ content (**d**) and SOD (**e**), CAT (**f**), POD (**g**) activities in WT and *TaNAC29*-overexpression (OE) lines under normal and 250 mM salt stress conditions. Seedlings leaves were sampled from WT and *TaNAC29*-overexpression lines at 0 (as a negative control), 10, 21 or 28 DAT to detect physiological indices. Values are means ± SE of three replicates. Asterisks indicate statistically significant differences from WT (*P < 0.05; **P < 0.01)

**Fig. 7:**
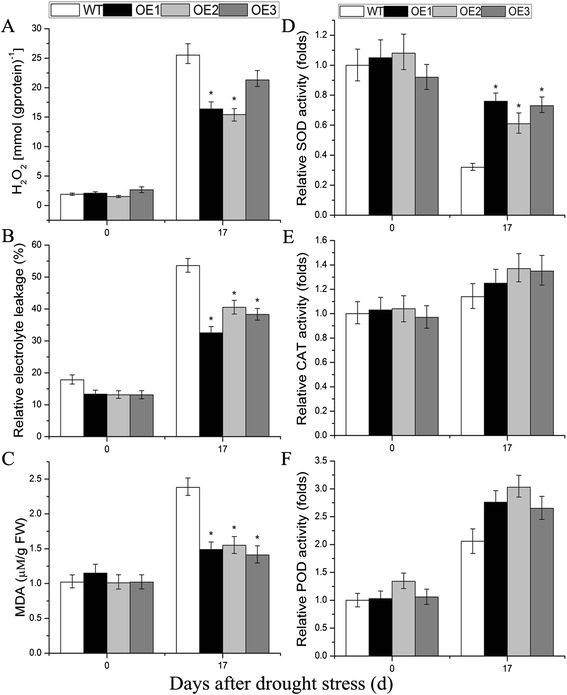
Analysis of physiological indices under drought stress conditions. Analysis of H_2_O_2_ content (**a**), electrolyte leakage (**b**), MDA content (**c**) and SOD (**d**), CAT (**e**), POD (**f**) activities in WT and *TaNAC29*-overexpression (OE) lines under normal and drought stress conditions. Seedlings leaves were sampled from WT and *TaNAC29*-overexpression lines at 0 (as a negative control) and 17 DAT to detect physiological indices. Values are means ± SE of three replicates. Asterisks indicate statistically significant differences from WT (*P < 0.05)

Abiotic stress can increase the accumulation of reactive oxygen species (ROS), leading to increased oxidative stress [34]. To estimate the level of abiotic stress damage to plant material, related physiological indices in WT and transgenic lines at different time points after stress treatment were measured. Under salt stress, the decrease in chlorophyll content was greater in WT plants than in *TaNAC29*-overexpression lines (Fig. 6a). There was no significant difference in H_2_O_2_ accumulation, relative electrolytic leakage, and MDA content between WT plants and *TaNAC29*-overexpressing lines under normal conditions (Figs. 6 and 7). However, under salt and drought stresses, WT plants showed a greater accumulation of H_2_O_2_ than transgenic lines at all time points. This demonstrated that WT plants were more seriously damaged, and overexpression of *TaNAC29* protected transgenic lines from abiotic stress. The electrolytic leakage of the WT plants and *TaNAC29*-overexpression lines increased significantly, though the increment of the WT was higher than observed in transgenic lines, suggesting increased membrane damage might lead to increased solute leakage. MDA content was significantly lower in *TaNAC29*-overexpression lines than in WT plants under stress conditions, indicating that the transgenic plants produced less ROS.

To further estimate antioxidant enzyme activities, the relative activities of CAT, SOD, and POD [35] in WT and *TaNAC29*-overexpression lines were measured at different time points following stress treatments. The enzyme activity of SOD in *TaNAC29*-overexpression plants was significantly higher than in WT plants after subjection to salt and drought stresses (Figs. 6e and 7d), suggesting more superoxide radicals were converted into O_2_ and H_2_O_2_ via SOD catalysis in transgenic plants [35]. CAT is an important antioxidant enzyme involved in H_2_O_2_ detoxification [35], levels decreased significantly in WT plants but only slightly changed in *TaNAC29*-overexpression lines following salt stress (Fig. 6f), indicating that *TaNAC29*-overexpression lines scavenged more H_2_O_2_. POD activity in WT plants and *TaNAC29*-overexpression lines significantly increased, yet the increment had no significant difference after salt and drought stresses (Figs. 6g and 7f). The higher antioxidant activity of enzymes in transgenic plants may lead to greater scavenging of ROS, thus increasing the survival rates of the transgenic plants [34, 35]. These results demonstrated that *TaNAC29*-overexpression lines increased resistance to salt and drought stresses through greater scavenging of ROS.

## Discussion

### *TaNAC29* plays important roles in abiotic stress response and senescence

The *TaNAC29*-overexpression plants had significantly increased tolerance to salt and drought stresses. Numerous studies show that NAC TFs play critical roles in the response to biotic and abiotic stresses [3]. *ATAF1*, *ANAC019*, *ANAC055*, and *RD26* all enhance tolerance to drought stress [4, 5]. Overexpression of *SNAC1*, *OsNAC5*, *OsNAC9*, *OsNAC6*, *OsNAC10*, and *OsNAC45* results in enhanced tolerance to abiotic stresses [9–15]. *TaNAC69* confers significant enhancement of drought tolerance in transgenic wheat [18]. Overexpression of *TaNAC2*, *TaNAC2a*, and *TaNAC67* in transgenic plants improves tolerance to multiple abiotic stresses [19–21]. The expression level of *TaNAC29* was much higher in senescent leaves, indicating that *TaNAC29* maybe also involved in leaf senescence. In *Arabidopsis*, *ANAC016*, *AtNAP/ANAC029*, *ATAF1*, and *ORE1/ANAC092* act as positive regulators of leaf senescence [30, 36–39], whereas, *JUB1* and *VNI2* act as negative regulators of leaf senescence [7, 8]; plants overexpressing these genes exhibit a delayed senescence phenotype [7, 8], similar to the *TaNAC29*-overexpression lines. Interestingly, these senescence associated NAC TFs are also involved in abiotic stresses. *ANAC016-*overexpression plants have low drought tolerance [40], and *JUB1-*overexpression lines have enhanced tolerance to salt and H_2_O_2_-induced oxidative stresses [7]. Thus, these senescence associated NAC TFs play important roles in the crosstalk between leaf senescence and the abiotic stress response, and it is likely that TaNAC29 has a similar role in wheat.

The overexpression of numerous genes enhances tolerance to abiotic stresses; and overexpression of key genes regulates the relative expression levels of other marker genes [33]. *RD29b* is an ABA-responsive marker gene in the SnRK2s pathway, and is induced by abiotic stress [29, 41]. Lu et al. [42] demonstrated that 35S-ABI1 transgenic *Arabidopsis* plants grow better than WT under high C/low N stress conditions, while *RD29b* relative expression in transgenic *Arabidopsis* is significantly lower than observed in WT plants under high C/low C conditions. Yang et al. [8] demonstrated that *RD29b* transcript levels gradually increased during leaf senescence, similar to *VNI2* expression. Our results of *RD29b* relative expression are similar to these reports. *SAG113* expression levels were significantly reduced in *TaNAC29*-overexpressing plants, suggesting that TaNAC29 is involved in the regulation of *SAG113* expression. Compared with WT plants, *SAG113* knockout plants show less water loss, more sensitive to ABA, delayed leaf senescence, and enhanced tolerance to drought stress [31]; this is similar to the *TaNAC29*-overexpression line plants. Thus, down-regulation of *SAG113* expression in plants overexpressing *TaNAC29* likely contributed to the enhanced salt and drought stresses tolerance observed.

### *TaNAC29* participates in the ABA-mediated pathway in stress tolerance

ABA is an important signaling molecule involved in plant responses to many unfavorable environmental stresses, including high salinity, drought, and extreme temperature [33], Indeed, ABA is often considered as the second messenger. Our results demonstrated that plants overexpressing *TaNAC29* had enhanced tolerance to salt and drought, and exhibited hypersensitivity to ABA. This increased sensitivity to ABA might be a result of lower transpiration rates and faster stomatal closure in *TaNAC29*-overexpression plants, leading to lower water loss and enhanced tolerance. A close relationship between ABA-hypersensitivity and abiotic stress tolerance has been demonstrated. For example, overexpression of *K*^*+*^*uptake transporter 6* (*KUP6*) in *Arabidopsis* exhibits increased ABA sensitivity through faster stomatal closure and enhanced tolerance to drought stress [43]. Conversely, mutation in *Arabidopsis more axillary growth 2* (*MAX2*), *MAX3*, and *MAX4* genes results in reduced ABA sensitivity, impaired ABA-mediated stomatal closing, and decreased survival rates under drought stress [44]. Numerous studies have revealed how abiotic stresses trigger signal transductions necessary for plant survival via ABA and other phytohormone signal molecules. In *Arabidopsis*, ATAF1 acts as a positive regulator of ABA biosynthesis [45]. Conversely, *ATAF2* expression is induced by methyl jasmonate (MeJA) and salicylic acid, but is independent of ABA signaling [6]. Plants overexpressing *ATAF1*, *ANAC019*, *RD26*, and *OsNAP* show enhanced tolerance to drought, but increased sensitivity to ABA [5, 28, 46, 47]. Overexpression of *ANAC019* and *ANAC055* increases JA-induced expression of defense genes [48]. *NTM2*/*ANAC069*-mediated salt signaling in seed germination is not related to ABA and gibberellin (GA), meaning NTM2 acts independently of the ABA signal [49]. *VNI2* is induced by high salinity in an ABA-dependent manner; this gene delays leaf aging and senescence [8]. Thus, many genes participating in the ABA signaling transduction network, such as PP2Cs, SnRK2s, NAC, and WRKY, are responsive to external influences [33]. Hence, the ABA hypersensitivity of *TaNAC29* suggests that *TaNAC29* is involved in cellular network cross-talk between the ABA signal pathway and the abiotic stress-induced metabolic pathway.

### The antioxidant mechanism is involved in *TaNAC29* conferring salt and drought stresses tolerance

Abiotic stress can lead to oxidation damage and membrane lipid peroxidation in plants [34, 35]. Therefore, the H_2_O_2_ content often increases under stress conditions. In *Arabidopsis*, H_2_O_2_ treatment increases *JUB1* and *ATAF1* transcript abundance [7, 30]. Plants overexpressing *JUB1* counteract cellular accumulation of H_2_O_2_, having decreased H_2_O_2_ levels and an enhanced tolerance to salt stress [7]. In contrast, *ATAF1*-overexpression plants accumulate significantly higher H_2_O_2_ than WT plants; *ataf1* mutant plants accumulate less H_2_O_2_ and display delayed senescence [30]. *TaNAC29*-overexpression plants also accumulated less H_2_O_2_ and had enhanced tolerance to salt and drought stresses. MDA is often increased under stress conditions, and ROS can be produced by membrane lipid peroxidation thus affecting protein synthesis and stability in plants [34, 35]. Hence, the extent of membrane lipid peroxidation was used to assess the severity of oxidation stress. Antioxidant enzymes such as CAT, SOD, and POD play an important role in scavenging ROS [34, 35]. These antioxidant enzymes are often upregulated to defend protein systems after stress, however, several studies have demonstrated that activities of these antioxidant enzymes decline under salt and drought stresses over long time treatment conditions, suggesting that the defense mechanism of these antioxidant enzymes may have been destroyed [34, 35]. Thus, together with the higher enzyme activities of SOD or CAT (Figs. 6 and 7), most *TaNAC29*-overexpression plants still survived under salt and drought stresses conditions.

## Conclusions

In this study, *TaNAC29* was upregulated by various abiotic stresses, and played important roles in senescence and tolerance to high salinity and drought. It was demonstrated that *TaNAC29* participates in the ABA-mediated pathway, and activates antioxidant enzymes to improve plant tolerances. These findings shed some light on the complex mechanisms and role of NAC in a plant’s response to environmental stresses.

## Methods

### Plant materials and stress treatments

Bread wheat (*Triticum aestivum* L. cv. Chinese spring) was used in this study. Seeds were germinated in water and cultivated under a 12-h light/12-h dark cycle at 22 °C in a greenhouse. Fourteen-day-old wheat seedlings were treated by different stresses or signaling molecules. Multiple abiotic stress treatments were performed by submerging wheat seedling roots in solutions of 200 mM NaCl, 20 % PEG6000, and 10 mM H_2_O_2_, respectively. For phytohormone treatments, wheat seedling leaves were sprayed with 100 μM ABA, meanwhile wheat seedling roots were submerged in the same concentration of ABA solution. Wheat seedlings treated with water were used as a mock control. Leaves and roots of wheat seedlings were sampled at different time-points (0, 1, 3, 6, 12, and 24 h). Field grown wheat plants were used for measuring organ-specific expression patterns of *TaNAC29*. Young root, stem, and leaf (at the four-leaf stage), mature root, stem, and leaf (at anthesis stage), flag leaf, stamen, pistil, embryo, endosperm, coleoptile, and caryopsis were collected. All collected samples were immediately frozen in liquid nitrogen and stored at −80 °C for future analysis.

### Cloning and bioinformatic analysis of *TaNAC29*

To identify novel *NAC* genes in wheat, *in silico* cloning was used to predict putative *TaNAC* genes. Previously, Jeong et al. [11] reported that *OsNAC10*-overexpression rice plants had enhanced tolerance to drought stress. Therefore, the barley (*Hordeum vulgare*) gene *HvNAC023* (GenBank: CBZ41159.1), with high homology to *OsNAC10*, was used as a query probe to blast the EST library of wheat (http://www.ncbi.nlm.nih.gov/nucest/?term=Triticum+aesti vum). Some highly homologous EST sequences were obtained, and assembled using the CAP3 program (http://doua.prabi.fr/software/cap3). Next, the predicted NAC-like gene was used as a query probe to blast the EST library of wheat again, thus obtaining a longer sequence. After repeats for several times, a full-length cDNA sequence, including ORF, 5′-UTR, and 3′-UTR regions was obtained. Next, primer pairs (Additional file 2: Table S1a) were designed to amplify this putative sequence from cDNA templates synthesized from RNAs mixtures extracted from different wheat organs and under various stress conditions (see above). The polymerase chain reaction (PCR) product was cloned into a pMD18-T vector (TaKaRa, Dalian, China) and transfected into *E. coli* TOP 10 competent cells (Tiangen, Beijing, China). Finally, the target gene in positive cloned strains was sequenced (AuGCT Biotech, Beijing, China). The cloned *NAC* gene, designated *TaNAC29* (GenBank: KT783450), was analyzed using online software available at the InterProScan website (http://www.ebi.ac.uk/Tools/pfa/iprscan5/). The conserved NAC domain at the N-terminal, and the L motif (also named LP motif) [27, 28] at the C-terminal, were searched using MEME (http://meme-suite.org/tools/meme). A prediction of structural ID was performed using PONDR VL3 (http://www.pondr.com). Alignment of relevant sequences was performed using MEGA software (version 5.1) and MegAlign of DNAStar.

### qRT-PCR analysis of *TaNAC29* expression patterns

Total RNA was extracted from different organs and various stress treated materials (see above) using Trizol in accordance with manufacturer’s instructions (Zomanbio, Beijing, China). Integrity and quality of total RNA samples were examined by electrophoreses by running 5 μl RNA in an ethidium bromide agarose gel. First-strand cDNA synthesis was performed according to the FastQuant RT kit protocol (Tiangen). Organ-specific expression patterns and gene expression patterns of *TaNAC29*, after various stress treatments, were determined by qRT-PCR analysis. Gene specific primer pairs (Additional file 2: Table S1b) were designed at the 3′-UTR region of the nucleic acid sequence, thus excluding the NAC conserved domain. To test primer accuracy and specificity, PCR was performed and the amplified product was confirmed by sequencing. *TaActin* (GenBank: AB181991.1; Additional file 2: Table S1b) was used as the internal reference gene when examining gene expression of *TaNAC29*. qRT-PCR analysis was performed using a Biorad CFX system (Bio-Rad Laboratories, Hercules, CA). During the qRT-PCR analysis, each sample was analyzed using three technical replicates, and data analyzed by analysis software based on the comparative 2^−ΔΔCT^ method of relative gene quantification [50]. Relative expression of *TaNAC29* and probability value (*P*) were also calculated using the qRT-PCR analysis software.

### Subcellular localization of TaNAC29 in wheat protoplasts

To investigate the subcellular localization of TaNAC29, a recombinant construct of *TaNAC29-GFP* was transformed into wheat mesophyll protoplasts using the DNA-PEG-calcium transfection method [51]. To obtain high concentration recombinant plasmids, the pMD18-T plasmid was fused with *cauliflower mosaic virus* 35S promoter and GFP sequence, resulting in the transformation plasmid pMD18-35S-GFP vector. The *TaNAC29* ORF (excluding the termination codon), containing *Xba*I/*Sma*I restriction sites, was amplified using specific primer pairs (Additional file 2: Table S1c). The PCR product was then inserted into the *Xba*I/*Sma*I sites of the pMD18-35S-GFP vector. Subsequently, 35S::TaNAC29-GFP fusion protein construct, and the pMD18-35S-GFP vector as a negative control, were separately introduced into wheat mesophyll protoplasts using the DNA-PEG-calcium transfection method [51]. Finally, transformed mesophyll protoplasts were examined by fluorescence microscopy (OLYMPUS DP72, Japan) after incubation at 20 °C for 20 h in the dark. The specific nuclear stain DAPI (Beyotime Biotech, Jiangsu, China) was used for observing transformed mesophyll protoplasts.

### Transactivation activity analysis of TaNAC29 in yeast

Transactivation activity analysis was performed to examine the expressions of *HIS3*, *ADE2*, and *LacZ* reporter genes in the yeast strain *AH109*. The full-length ORF (TaNAC29_1–357_), N-terminal domain (TaNAC29_1–177_), C-terminal regulatory (TaNAC29_178–357_), TaNAC29_1–233_ without L motif and six truncated versions (TaNAC29_1–261_, TaNAC29_1–250_, TaNAC29_178–242_, TaNAC29_243–273_, TaNAC29_274–312_, and TaNAC29_313–357_) with particular L motif sequences [27] were amplified by PCR using specific primers containing *Eco*R1/*Bam*H1 restriction sites (Additional file 2: Table S1d). Each PCR product was inserted into *Eco*R1/*Bam*H1 sites of the pGBKT7 vector (Clontech, Palo Alto, CA). Subsequently, the ten constructs and negative control pGBKT7 vector were transformed into yeast strain *AH109* (Clontech). The transformed yeast cultures were plated onto synthetic dropout (SD) plates (−Trp) and SD plates (−Trp/-His/-Ade) for 4 d at 30 °C, before detection of transactivation property. For easy evaluation of transactivation activity, transformants were dissolved in sterile distilled water and 10-fold serial dilutions prepared. Finally, 3 μl of each dilution was spotted onto SD plates (−Trp/-His/-Ade) and SD plates (−Trp/-His) with X-α-Gal (Clontech) for 4 d at 30 °C.

### Plant transformation and generation of transgenic plants

To obtain transgenic *Arabidopsis* plants, the coding sequence of the *TaNAC29* containing termination codon was amplified by RT-PCR and cloned into *Xba*I/*Sma*I restriction sites of the pBI121 vector (Clontech) under the control of the 35S promoter of *cauliflower mosaic virus*. The primers containing the *Xba*I/*Sma*I restriction sites are listed in Additional file 2: Table S1e. The recombinant vector pBI121-TaNAC29 and the pBI121 empty VC were introduced into *Agrobacterium tumefaciens* strain *EHA105* (Biovector Science Lab, Inc., Beijing, China). Finally, transgenic *Arabidopsis* plants were generated using the *A. tumefaciens*-mediated floral dipping method [52]. For generation of homozygous progenies, T_1_ and T_2_ seeds were screened on kanamycin (50 mg/L) plates for selection. Homozygous T_3_ progenies were confirmed by PCR analysis, and *TaNAC29* expression levels were examined by semi-quantitative analysis using gene-specific primers (Additional file 2: Table S1e). Representative lines overexpressing *TaNAC29* were used for further analysis.

### Stress tolerance assays of *TaNAC29*-overexpression plants

The WT, VC, and TaNAC29-overexpression lines were used to evaluate various stress tolerances. After stratification at 4 °C for 3 d, *Arabidopsis* seeds were germinated on 1/2 MS medium (1 % sucrose, no vitamins) under a 16-h light/8-h dark cycle at 22 °C in a growth chamber for an additional 4 d. Four-day-old seedlings were transferred to 1/2 MS medium supplemented with 10 μM ABA for signal molecule response assay, and with 120 mM NaCl or 400 mM Mannitol for osmotic stress assays. Root length was scored after 8 or 16 d of growth on vertical plates. To conduct seedling emergence rate assays for inspection of the ABA response, *Arabidopsis* seeds were sown on 1/2 MS medium supplemented with 0 or 2 μM ABA. Seedling emergence rates (seedling with cotyledon) were scored on plates after 8 d growth.

To obtain full-grown *Arabidopsis* plants, WT, VC, and *TaNAC29*-overexpression plants were grown in plastic containers filled with humus soil and cultivated in a greenhouse with a 16-h light (80 or 120 μmol m^−2^ s^−1^)/8-h dark cycle at 22 °C. For the water loss assay, rosette leaves were detached from 25-day-old seedlings and placed on filter paper for water deficiency treatment at 25 °C. Meanwhile changes in rosette leaf shape were observed during the air-dry treatment. The weight of rosette leaves was measured at 1-h intervals. Drought tolerance assays were performed, at different growth periods (25-, and 65-day-old), using *Arabidopsis* plants cultured in a greenhouse without watering. For the salt tolerance assay, *Arabidopsis* plants grown at different growth periods (25-, 45-, and 65-day-old) were subjected to 250 mM NaCl treatment for 21 or 28 d after withholding of water for 2 w.

### Expression analyses of relevant marker genes

To detect expression of relevant marker genes in WT and *TaNAC29*-overexpression plants under salt and drought stresses, leaves of *Arabidopsis* seedlings were detached after subjection to 250 mM salt stress for 10 d or drought stress for 17 d. Total RNA was extracted from leaves using Trizol in accordance with manufacturer’s instructions (Zomanbio, Beijing, China), and qRT-PCR analysis performed to examine marker gene expressions of *RD29b* (*At5g52300*), *ERD11* (*At1g02930*), *AIB1* (*At2g46510*), *ABI5* (*At2g36270*), *SAG13* (*At2g29350*), and *SAG113* (*At5g59220*); *Actin2* was used as the internal control gene. Three independent biological experiments were performed. Primer pairs used are listed in Additional file 2: Table S1f.

### Measurement of electrolytic leakage, of chlorophyll, MDA, and H_2_O_2_ content, and of SOD, CAT, and POD activity

To detect changes in physiological indices under salt and drought stress conditions, leaves were collected from plants at different time points (0, 10, 17, 21, and 28 d) during stress treatments. Electrolytic leakage, the contents of chlorophyll, MDA, and H_2_O_2_, and the enzyme activities of SOD, CAT, and POD were measured. For extraction of H_2_O_2_, CAT, SOD, and POD, 0.2 g of leaves were sampled with 1.8 ml phosphate-buffered saline (PBS; 0.1 mol/L, pH 7.4) on ice. The crude extract was centrifuged at 10,000 *g* for 10 min at 4 °C. The H_2_O_2_ content, and CAT, SOD, and POD enzyme activities in the supernatant were immediately measured by enzyme-linked immunosorbent assay (ELISA), using the corresponding detection kits (A064-1, A007-1, A001-1, and A084-3; Jiancheng, China). MDA content analysis was performed using the thiobarbituric acid method [53]. Chlorophyll was extracted and examined using the protocol [54]. Electrolyte leakage was determined by relative conductivity as described by Hu [55].

### Availability of supporting data

All the supporting data are included as additional files.

## Additional files

Additional file 1: Figure S1. Structural features of TaNAC29 protein. **Figure S2.** Phylogenetic relationship of TaNAC29 with other NAC members. **Figure S3.** Alignment of the nucleic acid sequences. **Figure S4.** Transactivation activity analysis of TaNAC29 protein in yeast. **Figure S5.** Subcellular localization of TaNAC29. **Figure S6.** Organ-specific expression patterns of *TaNAC29* in wheat. **Figure S7.** Expression of *TaNAC29* in transgenic *Arabidopsis* lines. **Figure S8.** Delaying in bolting and flowering of *TaNAC29-*overexpression line 1 (OE1) in comparison to the WT plants. **Figure S9.** *TaNAC29* overexpression (OE) lines exhibit enhanced tolerance to water deficiency. **Figure S10.** Root growth of WT and *TaNAC29*-overexpression (OE) lines in response to drought stress. (PDF 1392 kb) Additional file 2: Table S1. Primers used in amplification of DNA sequences. (PDF 83 kb)

## Abbreviations

ABA: Abscisic acid
*ABI5*: ABA-insensitive 5
*AIB1*: ABA-inducible BHLH-type transcription factor/JA-associated MYC2-like1
CAT: Catalase
d: Day
DAPI: 4′,6-diamidino-2-phenylindole
ELISA: Enzyme-linked immunosorbent assay
*ERD11*: Early-responsive-to-dehydration 11
GA: Gibberellin
GFP: Green fluorescent protein
ID: Intrinsically disordered
JA: Jasmonic acid
*KUP6*: *K*^*+*^*uptake transporter 6*
*MAX2*: *More axillary growth* 2
MDA: Malondialdehyde
MeJA: Methyl jasmonate
MEME: Multiple EM for motif elicitation
MS: Murashige-Skoog
NAC: NAM, ATAF and CUC
OE: Overexpression
ORF: Open reading frame
*P*: Probability value
PBS: Phosphate-buffered saline
PCR: Polymerase chain reaction
POD: Peroxidase
qRT-PCR: Quantitative real-time polymerase chain reaction
*RD29b*: Responsive-to-desiccation 29b
ROS: Reactive oxygen species
*SAG13*: Senescence-associated gene 13
*SAG113*: Senescence-associated gene 113
SD: Synthetic dropout
SOD: Superoxide dismutase
TFs: Transcription factors
UTR: Untranslated region
VC: Vector control
w: Week
WT: Wild type
