# Supplementary material for: TaNAC29, a NAC transcription factor from wheat, enhances salt and drought tolerance in transgenic Arabidopsis
Source: BMC Plant Biol. 2015 Nov 4;15:268. doi: 10.1186/s12870-015-0644-9 (PMC4632686; doi:10.1186/s12870-015-0644-9)
Supplement: Additional file 1: Figure S1. — Structural features of TaNAC29 protein. Figure S2. Phylogenetic relationship of TaNAC29 with other NAC members. Figure S3. Alignment of the nucleic acid sequences. Figure S4. Transactivation activity analysis of TaNAC29 protein in yeast. Figure S5. Subcellular localization of TaNAC29. Figure S6. Organ-specific expression patterns of TaNAC29 in wheat. Figure S7. Expression of TaNAC29 in transgenic Arabidopsis lines. Figure S8. Delaying in bolting and flowering of TaNAC29-overexpression line 1 (OE1) in comparison to the WT plants. Figure S9. TaNAC29 overexpression (OE) lines exhibit enhanced tolerance to water deficiency. Figure S10. Root growth of WT and TaNAC29-overexpression (OE) lines in response to drought stress. (PDF 1392 kb) [file 12870_2015_644_MOESM1_ESM.pdf]

TaNAC29, a NAC transcription factor from wheat, enhances salt and drought tolerance in transgenic *Arabidopsis*. Quanjun Huang, Yan Wang, Bin Li, Junli Chang, Mingjie Chen, Kexiu Li, Guangxiao Yang and Guangyuan He.

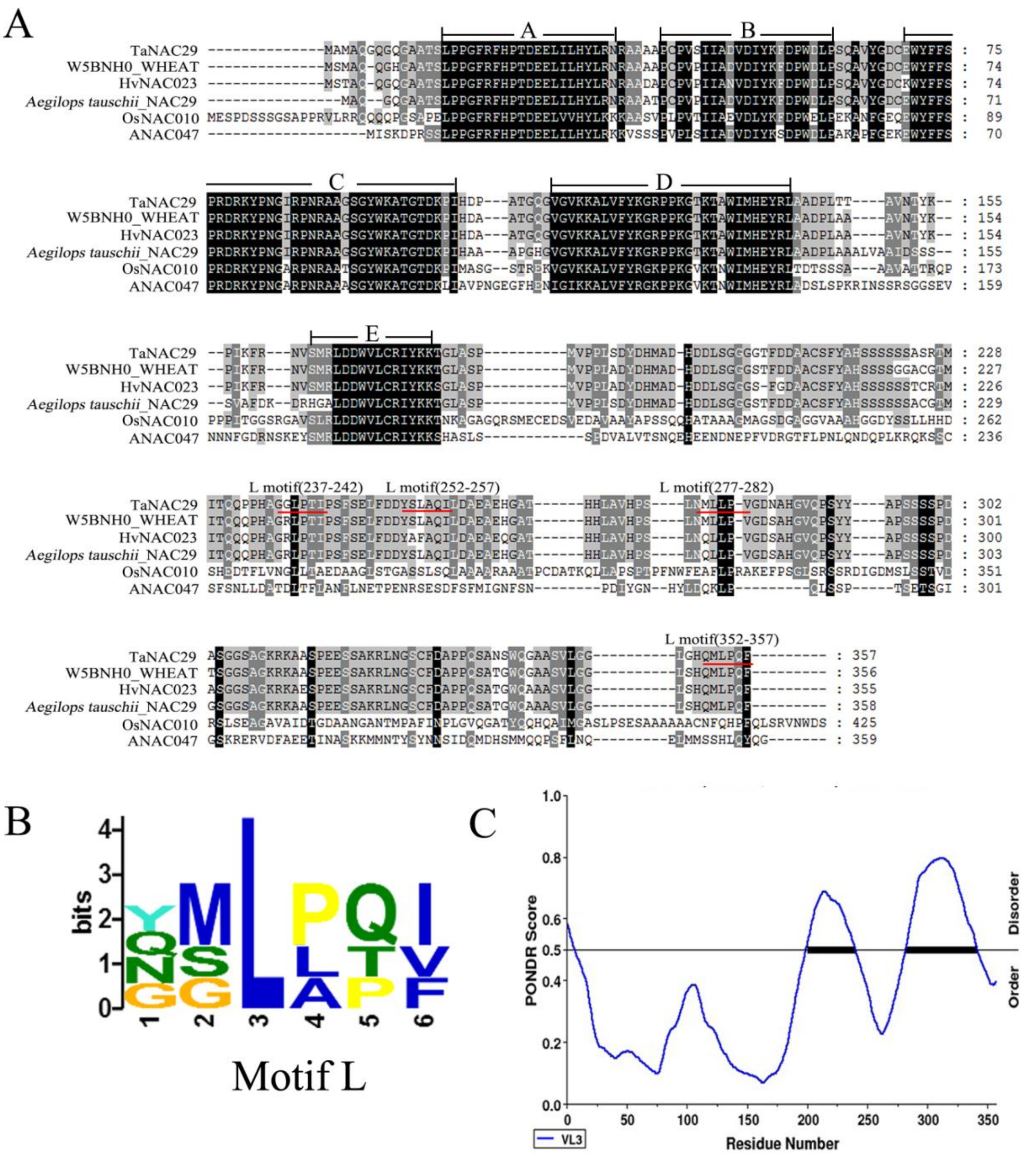

Figure S1. Structural features of TaNAC29 protein

(A) Multiple alignment of amino acid sequences of TaNAC29 and other related NAC proteins from selected plant species. Alignments were performed using MEGA5.1 program. L motif was indicated by the red bar after MEME searching. Five conserved NAC-subdomain were indicated.

(B) Weblogo of L motif.

(C) PONDR VL3 analysis for ID of TaNAC29.

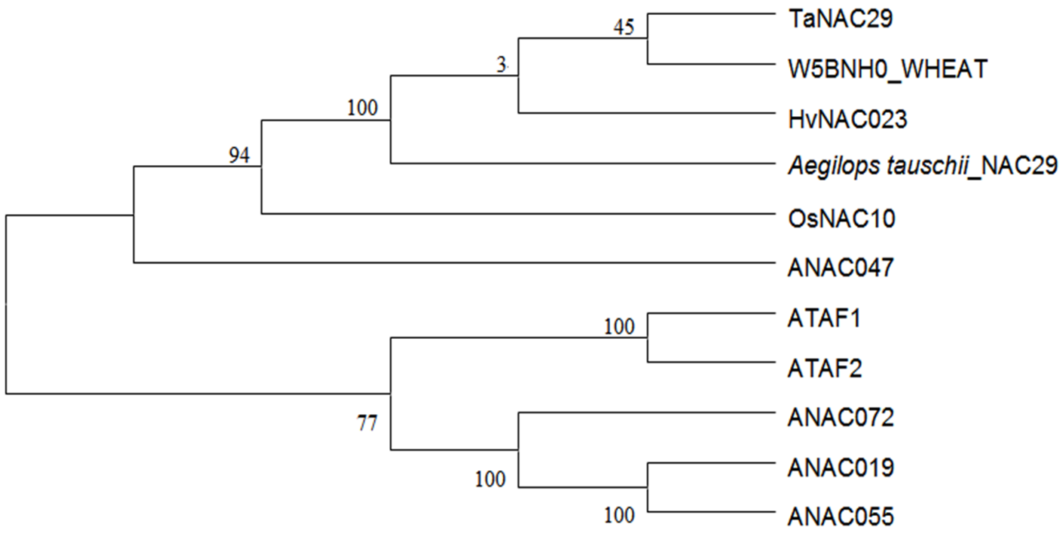

Figure S2. Phylogenetic relationship of TaNAC29 with other NAC members. Phylogenetic relationship of TaNAC29 with W5BNH0 (EMBL: C116E5668.1) from *Triticum aestivum*, HvNAC023 (GenBank: CBZ41159.1) from *Hordeum vulgare*, NAC29 (GenBank: EMT28859.1) from *Aegilops tauschii*, OsNAC10 (GenBank: EAZ40329.1) from *Oryza sativa*, ANAC047 (AT3G04070), ATAF1 (AT1G01720), ATAF2 (AT5G08790), ANAC072 (AT4G27410), ANAC019 (AT1G52890) and ANAC055 (AT3G15500).

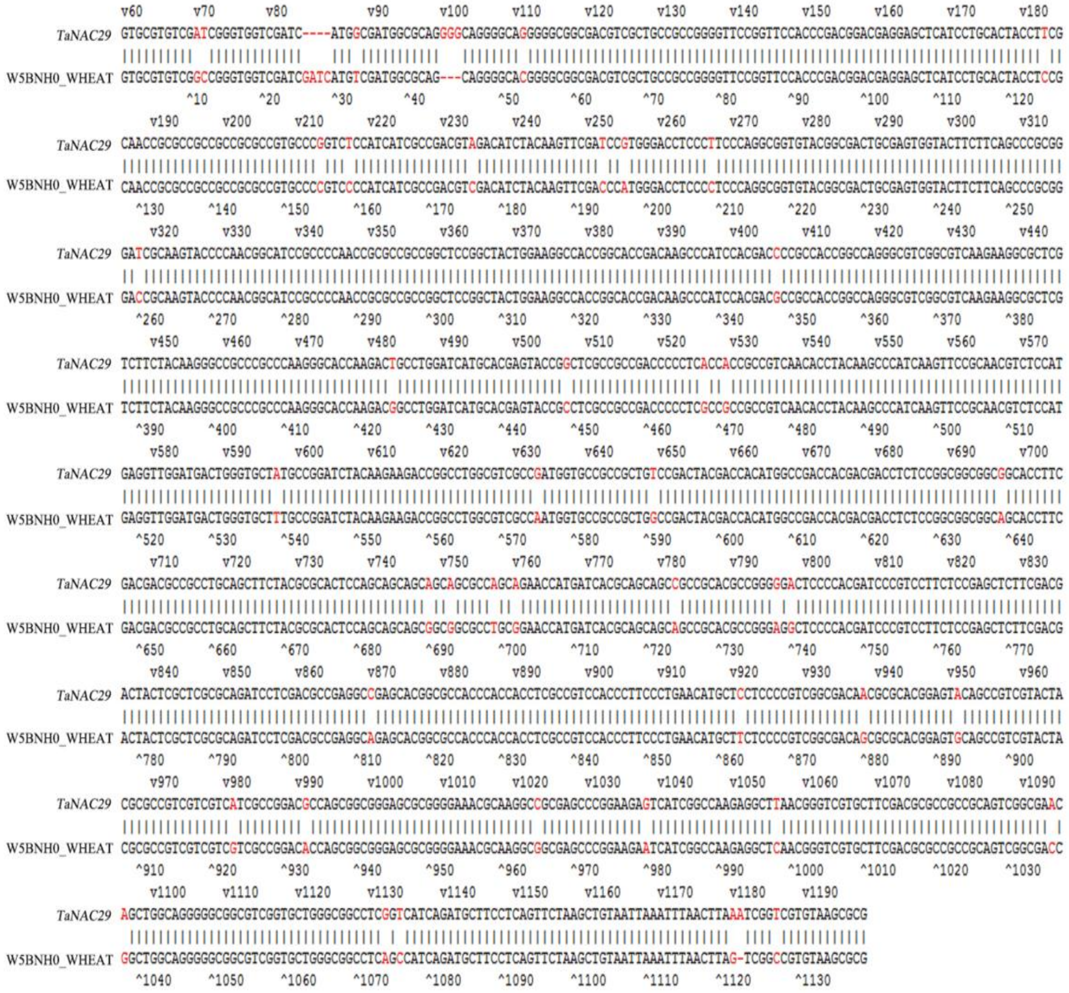

Figure S3. Alignment of the nucleic acid sequences. Alignment of the nucleic acid sequences of *TaNAC29* and W5BNH0_WHEAT. Alignments were performed using MegAlign of DANStar program.

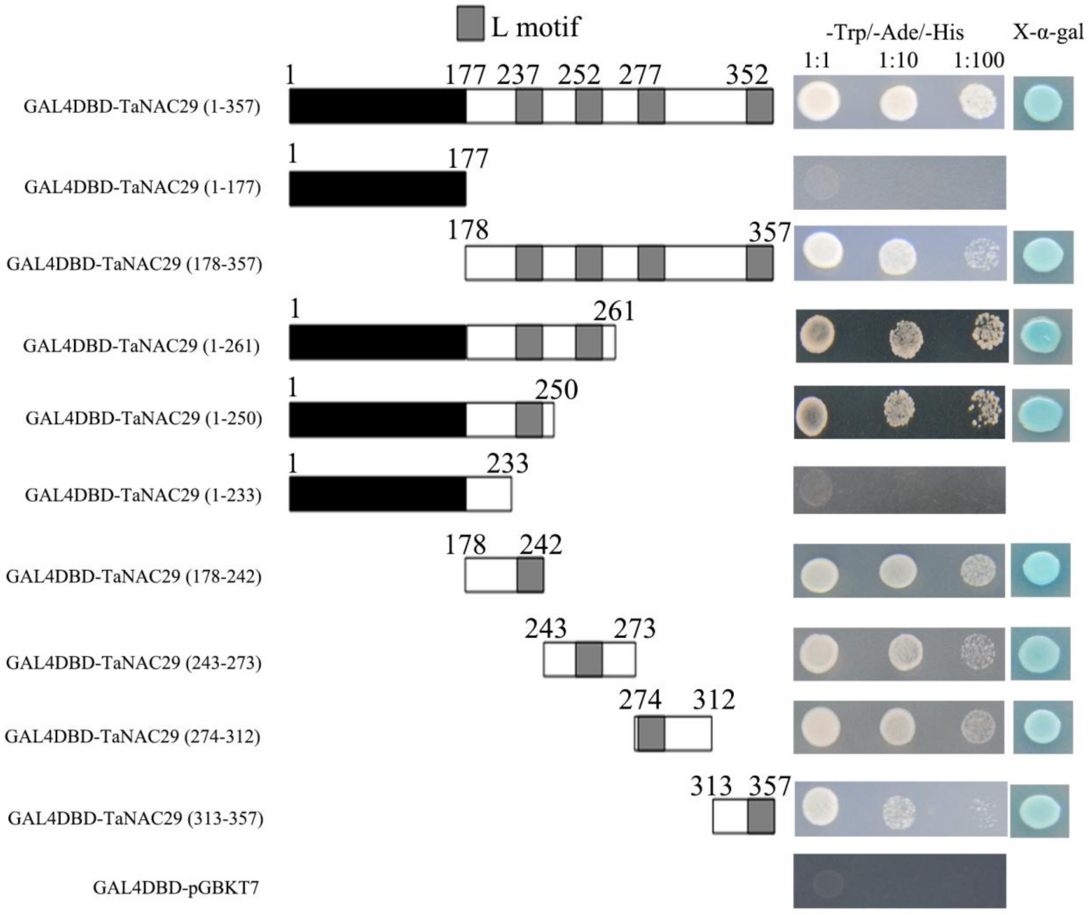

Figure S4. Transactivation activity analysis of TaNAC29 protein in yeast. Fusion proteins were expressed in yeast strain *AH109*, and then analysed for their transactivation activity of the *HIS3*, *ADE2* and *LacZ* reporter genes. Gal4-(DBD)-pGBKT7 served as negative control. Left, schematic diagram of full-length and truncated versions. A black bar indicates NAC domain, a gray bar indicates L motif. Right, transactivation activity assay.

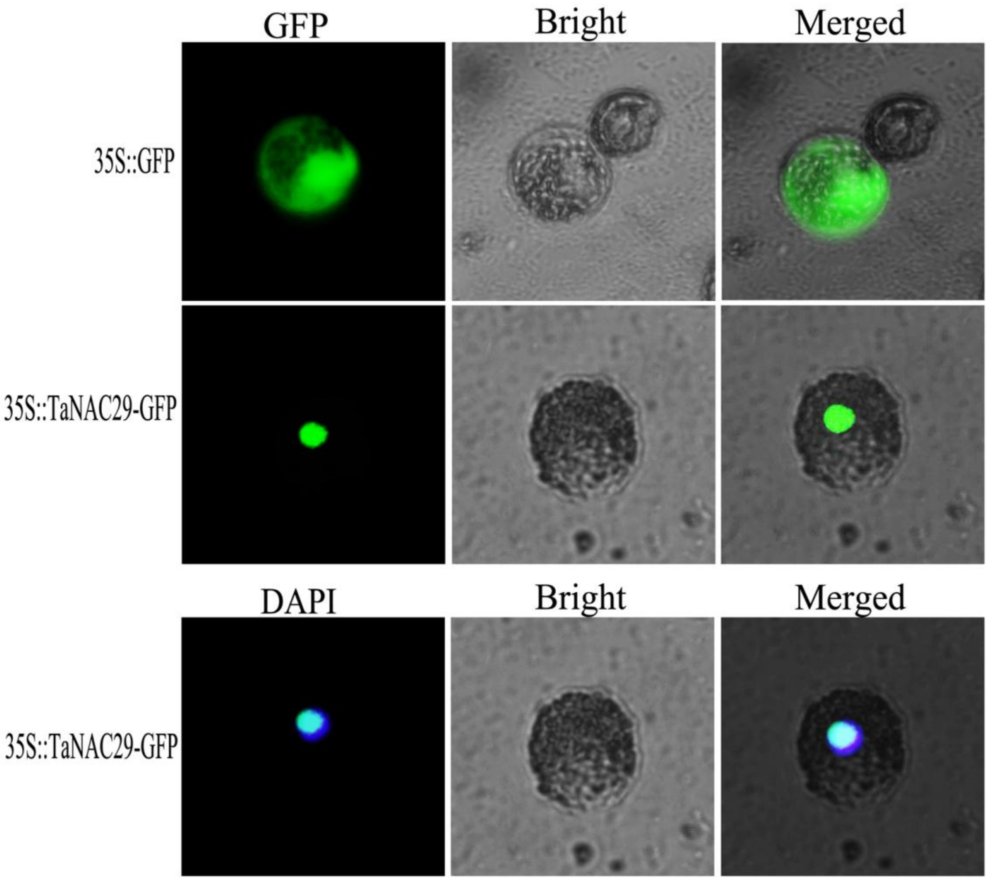

Figure S5. Subcellular localization of TaNAC29. The fusion protein 35S::*TaNAC29*-GFP and 35S::GFP (control) constructs were transiently expressed in wheat mesophyll protoplasts and visualized with fluorescence microscope. Top, 35S::GFP (control); middle, 35S::*TaNAC29*-GFP; bottom, 35S::*TaNAC29*-GFP+DAPI.

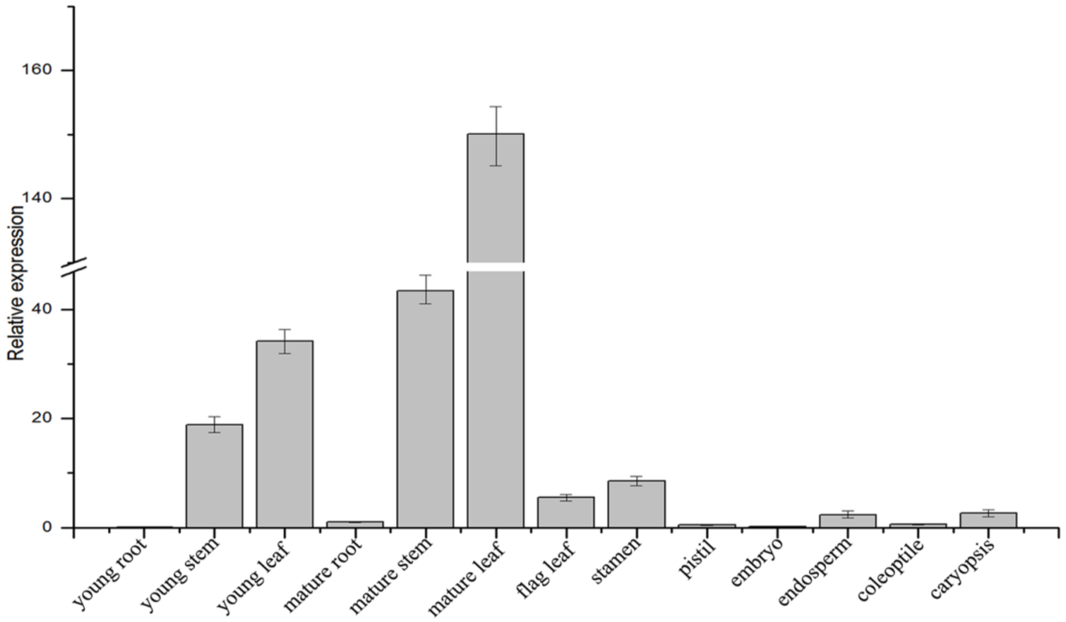

Figure S6. Organ-specific expression patterns of *TaNAC29* in wheat. Different organs include young root (at four-leaf stage); young stem (at four-leaf stage); young leave (at four-leaf stage); mature root (at anthesis stage); mature stem (at anthesis stage); mature leave (at anthesis stage); flag leaf; stamen; pistil; embryo; endosperm; coleoptile; caryopsis. The $2^{-\triangle\triangle CT}$ method was used in qRT-PCR analysis. Values are means ±SE of three replicates.

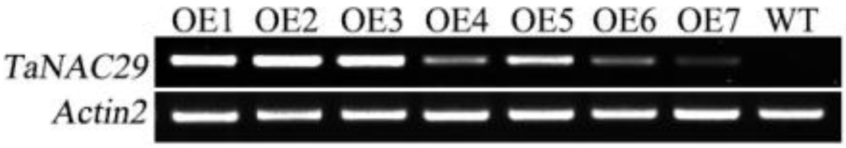

Figure S7. Expression of *TaNAC29* in transgenic *Arabidopsis* lines. The WT and transgenic *Arabidopsis* lines were cultured in 1/2 MS medium for two weeks and the whole seedlings were used to extract total RNA. *Actin2* as the internal control gene was used to examine the gene overexpression (OE) of *TaNAC29*. Three independent biological experiments were performed.

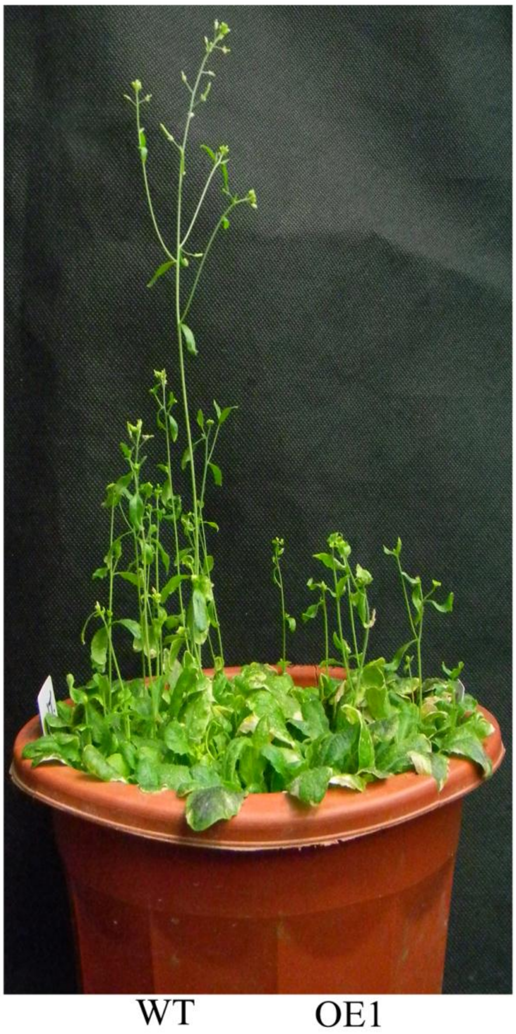

Figure S8. Delaying in bolting and flowering of *TaNAC29*-overexpression line 1 (OE1) in comparison to the WT plants.

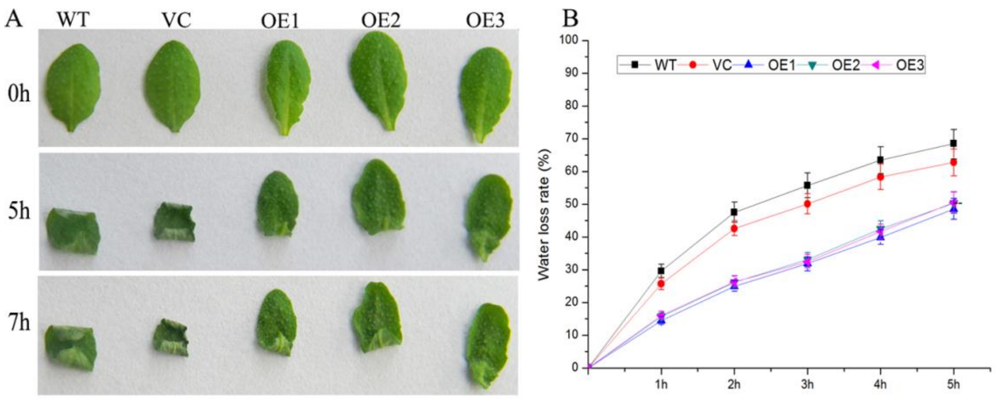

Figure S9. *TaNAC29* overexpression (OE) lines exhibit enhanced tolerance to water deficiency.

(A) Phenotypes of detached leaves under water deficiency treatment for 0 h, 5 h, and 7 h.

(B) Comparison of water loss rate. Values are means ± SE of three independent experiments.

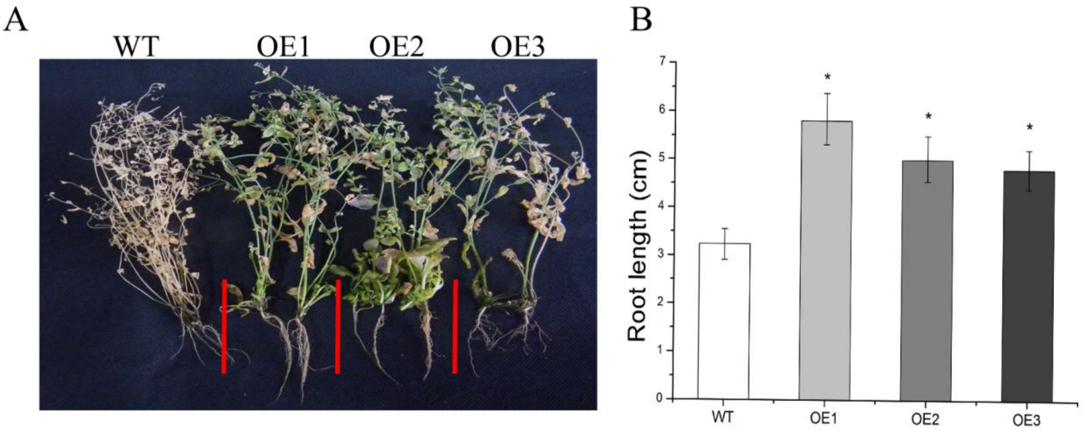

Figure S10. Root growth of WT and *TaNAC29*-overexpression (OE) lines in response to drought stress.

(A) Comparison of root length of WT and *TaNAC29*-overexpressing lines (65-day-old seedling) after drought stress.

(B) Quantitative analysis of root length. Values are means ± SE (n = 10 to 15 plants). Asterisks indicate statistically significant differences from WT (*$P < 0.05$).
