# Supplementary material for: TaNAC29, a NAC transcription factor from wheat, enhances salt and drought tolerance in transgenic Arabidopsis
Source: BMC Plant Biol. 2015 Nov 4;15:268. doi: 10.1186/s12870-015-0644-9 (PMC4632686; doi:10.1186/s12870-015-0644-9)
Supplement: Additional file 2: Table S1. — Primers used in amplification of DNA sequences. (PDF 83 kb) [file 12870_2015_644_MOESM2_ESM.pdf]

Table S1. Primers used in amplification of DNA sequences

(a) Primers used for cloning full length cDNA of *TaNAC29* from wheat

Gene Name	Forward/reverse primers
TaNAC29	5'- CTCCCTTCTTCTGATCGTGTG-3'
	5'- CGCGCTTACACGACCGATT-3'

(b) Primers used for RT-qPCR analysis of organ-specific and gene expression profiles of *TaNAC29*

Gene Name	Forward/reverse primers
TaNAC29	5'- CGAGCCCGGAAGAGTCAT-3'
	5'- CGCGCTTACACGACCGAT-3'
TaActin	5'- TCTATTTTGGCCTCTCTTAGCAC -3'
	5'- TTTCCTGTACCCCTTATTCCTC -3'

(c) Primers used for cloning of TaNAC29-GFP fusion protein for testing subcellular localization

Gene Name	Forward/reverse primers	Restriction enzyme
TaNAC29	5'- TGCTCTAGAATGGCGATGGCGCAGGG -3'	*Xba*I
	5'- TCCCCCGGGGAACTGAGGAAGCATC -3'	*Sma*I

(d) Primers used for cloning complete and truncated *TaNAC29* for their transactivation analysis

GAL4DBD–NAC	Forward/reverse primers	Restriction enzymes
$TaNAC29_{1\text{-}357}$	5'- CCGGAATTCATGGCGATGGCGCAGG-3'	*Eco*R1
	5'- CGCGGATCCTTAGAACTGAGGAAGCAT-3'	*Bam*H1
$TaNAC29_{1\text{-}177}$	5'- CCGGAATTCATGGCGATGGCGCAGG-3'	*EcoR*1
	5'- CGCGGATCCCTTCTTGTAGATCCGGCAT-3'	*Bam*H1
$TaNAC29_{178\text{-}357}$	5'- CCGGAATTCACCGGCCTGGCGTCGCCGAT-3'	*Eco*R1
	5'- CGCGGATCCTTAGAACTGAGGAAGCAT-3'	*Bam*H1
$TaNAC29_{1\text{-}261}$	5'- CCGGAATTCATGGCGATGGCGCAGG-3'	*Eco*R1
	5'-CGCGGATCCCTCGGCGTCGAGGATCTG-3'	*Bam*H1
$TaNAC29_{1\text{-}250}$	5'- CCGGAATTCATGGCGATGGCGCAGG-3'	*Eco*R1
	5'- CGCGGATCCGTCGAAGAGCTCGGAGAAG-3'	*Bam*H1
$TaNAC29_{1\text{-}233}$	5'- CCGGAATTCATGGCGATGGCGCAGG-3'	*Eco*R1
	5'- CGCGGATCCCGGCTGCTGCGTGATCATGGT-3'	*Bam*H1
$TaNAC29_{178\text{-}242}$	5'- CCGGAATTCACCGGCCTGGCGTCGCCGAT-3'	*EcoR*1
	5'- CGCGGATCCGATCGTGGGGAGTC-3'	*Bam*H1
$TaNAC29_{243\text{-}273}$	5'- CCGGAATTCCCGTCCTTCTCCGAGCTCTTC-3'	*EcoR*1
	5'- CGCGGATCCGTGGACGGCGAGGTGGTGGGT-3'	*Bam*H1
$TaNAC29_{274\text{-}312}$	5'- CCGGAATTCCCTTCCCTGAACATGCTCCTC-3'	*Eco*R1
	5'- CGCGGATCCCTTGCGTTTCCCCGCGCTCCC-3'	*Bam*H1
$TaNAC29_{313\text{-}357}$	5'- CCGGAATTCGCCGCGAGCCCGGAAGAGTCA-3'	*Eco*R1
	5'- CGCGGATCCTTAGAACTGAGGAAGCAT -3'	*Bam*H1

(e) Primers used for cloning *TaNAC29* into pBI121 vector, and for semi-quantitative RT-PCR analysis

	Forward/reverse primers	Restriction enzymes
pBI121-TaNAC29	5'- TGCTCTAGAATGGCGATGGCGCAGGG-3'	*Xba*I
	5'- TCCCCCGGGTTAGAACTGAGGAAGCATC -3'	*Sma*I
semi-quantitative	5'- TTCGCGCTGATACCAGACGTT -3'	
RT-PCR analysis	5'- CCTTCCCTGAACATGCTCCT -3'	

(f) Primers used for analyzing expression of relevant genes in wild-type and *TaNAC29* transgenic lines by qRT-PCR method

Gene name	Forward/reverse primers
actin2	5'- GGTAACATTGTGCTCAGTGGTGG-3'
	5'- AACGACCTTAATCTTCATGCTGC-3'
RD29b	5'- GGAGTTCAAGATTCTGGGAAC-3'
	5'- CATCAAAGTTCACAAACAGAGGC-3'
SAG13	5'- AGGGAGCATCGTGCTCATATCC-3'
	5'- CCAGCTGATTCATGGCTCCTTTG-3'
SAG113	5'- ATTTTCTTATTCTCGCAAGTGAC-3'
	5'- AAACACATTCGAACGACGCTA-3'
ERD11	5'- CAACTAAGAAACTCTTCGAC-3'
	5'- ATTCAAATCAAACACTCGG-3'
AIB1	5'- TTGCGGGATCATCAGTTGCT-3'
	5'- TCCTGCTCCTGCTAACAAGC-3'
ABI5	5'- AACGGGAGATTGCGGACATT-3'
	5'- ACAGGGAACACTAGTAAAGCAGA-3'
